# Response to LTE “Bridging the Gap Between Patient Experience and System‐Level Review: A Call for Discipline‐Specific Communication in Hospital‐to‐Home Transitions

**DOI:** 10.1111/hex.70744

**Published:** 2026-06-24

**Authors:** J. van Grootel, R. Collet, M. Major, S. Wiertsema, J. van Dongen, M. van der Leeden, E. Geleijn, R. Ostelo, M. van der Schaaf

**Affiliations:** ^1^ Amsterdam Universitair Medische Centra Amsterdam the Netherlands; ^2^ Amsterdam Movement Sciences Amsterdam the Netherlands

Dear Editor,

We would like to thank the authors for their thoughtful response to our article, ‘Engaging patients in designing a transmural allied health pathway: A qualitative exploration of hospital‐to‐home transitions’, and for further advancing the discussion on communication during hospital‐to‐home transitions.

We agree with the authors that clear communication between all involved allied healthcare professionals across care settings is a prerequisite for seamless hospital‐to‐home transitions and that communication needs and barriers can differ between allied healthcare disciplines. While the authors observed that information from physical therapists is often more readily incorporated into generic discharge communication across care settings, we believe that this depends on the clinical context. Within our own experiences in developing, implementing and evaluating a transmural allied healthcare pathway, we observed that the involvement of different allied healthcare disciplines in discharge planning varied across clinical settings. For example, within neurological wards, occupational therapists and speech‐language therapists are often more structurally embedded within discharge planning processes compared with other hospital wards. These differences in involvement are likely to influence the extent to which discipline‐specific information is incorporated into generic discharge communication. Nevertheless, we believe that all allied healthcare disciplines can make an important contribution to safe and effective hospital‐to‐home transitions, as also emphasised by the authors.

The authors further contribute to the discussion by proposing several practical strategies to strengthen discipline‐specific communication during hospital‐to‐home transitions, including discipline‐specific checkboxes in discharge summaries, verbal handovers between hospital‐based and primary care professionals, and structured feedback mechanisms across care settings. In our view, these strategies all point toward the broader importance of interprofessional collaboration between allied healthcare professionals across care settings [1, 2]. Effective interprofessional collaboration requires not only the exchange of discipline‐specific information, but also a shared understanding of each other's roles, goals, and interventions, enabling care to be aligned and coordinated throughout hospital‐to‐home transitions. For example, whether discipline‐specific checkboxes are incorporated into discharge summaries partly depends on how allied healthcare professionals are embedded within multidisciplinary teams and whether their expertise is actively considered during discharge planning on the ward. Similarly, direct verbal handovers across care settings may become more feasible when professionals know each other's roles, expertise, and local contexts, facilitating more direct and timely communication [3]. This is consistent with our own observations that established relationships between hospital and geriatric rehabilitation professionals, and familiarity with each other's roles and working contexts, can facilitate communication and coordination during transitions of care. In addition, sustained collaboration across settings can facilitate regular evaluation of transitional care processes and help identify recurring barriers in communication and coordination. However, achieving and maintaining such collaboration remains challenging, particularly in primary care following discharge from hospital or rehabilitation settings, where professionals often work across organisational boundaries and have limited opportunities for direct contact and shared care planning.

In our experience, strengthening communication during hospital‐to‐home transitions requires recognition that transitional care takes place within a complex system in which multiple factors across care settings interact [4, 5]. Although interprofessional collaboration between allied healthcare professionals is an important component of transitional care, sustainable collaboration also depends on the environment in which professionals work. Organisational structures, available time for coordination, and access to technologies that support communication and information sharing across settings, such as shared electronic health records, also influence the extent to which collaboration can be integrated into routine practice. In addition, professionals across care settings often operate within separate organisational structures, priorities, and information systems, which can make shared ownership of transitional care processes difficult to establish and sustain. At the same time, greater attention to interprofessional collaboration within allied healthcare education can help prepare future professionals for the challenges of transitional care. Broader system‐level factors, including how transitional care activities and coordination efforts are recognised within healthcare financing structures, are likely to further shape opportunities for collaboration across settings. In this regard, when coordination, referral, and cross‐setting communication are not explicitly budgeted or reimbursed, they may remain dependent on individual professionals’ initiative rather than becoming a sustainable part of routine transitional care. Continued efforts at professional, organisational, educational, and system levels are therefore needed to further support effective communication and collaboration across allied healthcare disciplines during hospital‐to‐home transitions.

## Author Contributions

**J. van Grootel:** writing – original draft, validation, visualisation, writing – review and editing, conceptualisation, investigation. **R. Collet:** writing – review and editing. **M. Major:** writing – review and editing. **S. Wiertsema:** writing – review and editing. **J. van Dongen:** writing – review and editing. **M. van der Leeden:** writing – review and editing, supervision, validation. **E. Geleijn:** writing – review and editing. **R. Ostelo:** writing – review and editing. **M. van der Schaaf:** validation, writing – review and editing, supervision.

## Data Availability

Data sharing is not applicable to this article as no datasets were generated or analysed during the current study.
